# Hospital staff views of prescribing and discharge communication before and after electronic prescribing system implementation

**DOI:** 10.1007/s11096-017-0543-2

**Published:** 2017-10-26

**Authors:** Pamela Ruth Mills, Anita Elaine Weidmann, Derek Stewart

**Affiliations:** 1 Pharmacy Department, University Hospital Crosshouse, Kilmarnock, Ayrshire, Scotland KA2 0BE UK; 2 School of Pharmacy and Life Sciences, Robert Gordon University, Aberdeen, Scotland AB10 7GJ UK

**Keywords:** Behavioural determinants, Discharge communication, Hospital electronic prescribing and medicines administration, Patient safety, Qualitative research, Theoretical Domains Framework, United Kingdom

## Abstract

*Background* Electronic prescribing system implementation is recommended to improve patient safety and general practitioner's discharge information communication. There is a paucity of information about hospital staff perspectives before and after system implementation. *Objective* To explore hospital staff views regarding prescribing and discharge communication systems before and after hospital electronic prescribing and medicines administration (HEPMA) system implementation. *Setting* A 560 bed United Kingdom district general hospital. *Methods* Semi-structured face-to-face qualitative interviews with a purposive sample of hospital staff involved in the prescribing and discharge communication process. Interviews transcribed verbatim and coded using the Framework Approach. Behavioural aspects mapped to Theoretical Domains Framework (TDF) to highlight associated behavioural change determinants. *Main outcome measure* Staff perceptions before and after implementation. *Results* Nineteen hospital staff (consultant doctors, junior doctors, pharmacists and advanced nurse practitioners) participated before and after implementation. Pre-implementation main themes were inpatient chart and discharge letter design and discharge communication process with issues of illegible and inaccurate information. Improved safety was anticipated after implementation. Post-implementation themes were improved inpatient chart clarity and discharge letter quality. TDF domains relevant to staff behavioural determinants preimplementation were knowledge (task or environment); skills (competence); social/professional roles and identity; beliefs about capabilities; environmental context and resources (including incidents). An additional two were relevant post-implementation: social influences and behavioural regulation (including self-monitoring). Participants described challenges and patient safety concerns pre-implementation which were mostly resolved post-implementation. *Conclusion* HEPMA implementation produced perceptions of patient safety improvement. TDF use enabled behaviour change analysis due to implementation, for example, staff adoption of behaviours to ensure general practitioners receive good quality discharge information.

## Impacts on Practice

- Hospital electronic prescribing and medicine administration (HEPMA) system implementation results in improvements to hospital staff experience for prescribing and discharge communication
- After the implementation of a hospital electronic prescribing and medicine administration system, patient safety improvements are due to improved legibility and enhanced communication between secondary and primary care
- Hospital staff behaviour change amongst the different professional groups was evident as a direct consequence of HEPMA system implementation

## Introduction

Traditionally, hospital discharge information communication to patients’ general practitioners (GPs) in the United Kingdom (UK) has been provided by handwritten methods. This consisted of inpatient prescription review and manual transcription, with possible amendments, to another paper document, with addition of salient clinical information. Recent information technology (IT) developments have permitted introduction of new prescribing electronic systems into UK hospitals. The use of healthcare IT varies widely between nations and the UK lags behind other countries [1]. Within the European Union Sweden, Denmark and the Netherlands are cited as early adopters [2]. The European Association of Hospital Pharmacists (EAHP) called for “the universal application of electronic prescribing across Europe in order to deliver a step change in medication error prevention” [3]. UK policies now promote and advocate the use of electronic prescribing systems, with National Health Service (NHS) England aiming to have universal electronic systems by 2018 [4]. NHS Scotland eHealth strategy recommends hospital electronic prescribing and medicines administration (HEPMA) systems are implemented countrywide by end of 2017 [5]. Improved patient safety and GP communication are cited as core benefits of HEPMA implementation [6]. A 2009 NHS England report states that “e-prescribing systems will change how people work” and predicts HEPMA implementation will permit simple and direct discharge prescription production. However, it suggests hospital staff will develop “work-arounds” which may be helpful but may compromise safety [7].

Both NHS England and Scotland define the ideal content of discharge communication [8, 9]. Adopting discharge information guidance into clinical practice is essential to promote patient safety and continuity of care across the interface. The reported prevalence of discharge prescribing errors is extremely variable in part due to inconsistency in error definition and methods employed. A narrative literature review of discharge information communication and medicine discharge prescribing errors, which reviewed papers published between 2000 and 2014, reported discharge prescribing error prevalence ranging from 0.81 errors per patient to 17.5% medicines with errors [10]. This review highlighted a paucity of information relating to hospital staff perspectives before and experiences after implementation of innovative electronic solutions. Previous studies were mainly quantitative in design and tended to include an assessment of specific aspects of discharge communication, for example information content and accuracy [11–20]. The only study ascertaining opinions from hospital staff perspectives was reported by Yemm et al. [19] who invited junior hospital doctors (n = 74) to prioritise the content of discharge letters in a questionnaire survey. Qualitative research is less commonly reported, mainly ascertaining GPs’ opinions of the discharge communication process [12–15, 18, 20], but with little focus on the perspectives of hospital staff. There is therefore a need for further research into hospital staff perspectives, ideally using a qualitative approach to provide in-depth description and understanding prior to and following implementation.

HEPMA implementation may be considered a complex intervention, defined as containing several interacting components [21]. Complex intervention evaluation is described as difficult, “because of problems of developing, identifying, documenting, and reproducing the intervention” [22]. Focusing on behavioural determinants may aid evaluation type studies given that implementation deemed unsuccessful is associated with issues of behaviour, attitude, expectations and experience [22]. The Theoretical Domains Framework (TDF) was developed to identify key domains for successful healthcare intervention implementation with specific focus on behavior change interventions [23]. The TDF was developed from 33 theories of behaviour change synthesized into 14 domains of behavioural determinants. Furthermore, Cresswell and Sheikh propose that such studies should also consider exploring expectations and experiences [24].

### Aim of the study

The study aim was to describe health professionals’ perspectives involved in prescribing and discharge communication prior to and following HEPMA implementation and to examine associated behavioural determinants using the Theoretical Domains Framework (TDF) [23].

### Ethics approval

The study was approved by a UK university ethical review panel and NHS ethics committee advised ethical approval was not required.

## Method

### Study design

The study design used semi-structured face-to-face qualitative interviews to describe fully the perspectives of health professionals involved in discharge communication.

### Study setting

The study was undertaken in a 560 bedded UK district general hospital (DGH). Services provided include general medicine, general surgery, orthopaedic, gynaecology, oncology, maternity and paediatric inpatient wards. A HEPMA system was implemented at the study hospital between October 2013 and September 2014, with interviews completed between February and August 2013 (pre) and April to June 2015 (post).

### Sample size

It was anticipated prior to initiating interviews, that to achieve total population data saturation, a sample of five to six members of each professional group would be sufficient. If necessary, this number could be amended to achieve overall data saturation and not necessarily for each individual professional group.

Data saturation is defined as “the point in data collection when no new additional data are found that develops aspects of a conceptual category” and Francis et al. [25] claim it is essential to reach data saturation to ensure achievement of trustworthiness. The principles should be agreed by the research team prior to study commencement so that consensus may be reached about when to stop [25].

### Study participants

Included participants were members of identified staff groups, who worked at specified DGH, and were involved in the discharge communication process. A purposive stratified sampling approach was used. Service leads for consultant medical staff, junior medical staff, advance nurse practitioners and pharmacists were each asked to nominate five to six staff members. The aim was to recruit a diverse sample in terms of gender and years worked at the study setting. The length of time an individual had worked in the organisation may impact on their perceptions of systems and identified problems. More junior staff may be less aware of process and procedural problems. All nominated staff responded positively to the request and were provided with a participant information sheet and consent form by the principal researcher.Wherever possible, staff interviewed in the pre-implementation phase were re-interviewed. Immediately prior to interview the participant signed the consent form.

### Data generation

An interview schedule was developed based upon a narrative literature review undertaken by the research team [10], review of local medicine incident reports and consideration of Scottish Intercollegiate Guidelines Network (SIGN) 128 guideline recommendations [9]. Questions about both inpatient and discharge prescribing were included because any inpatient prescribing errors may be transferred on discharge. The schedule consisted of five sections: inpatient prescribing; discharge prescribing; the discharge communication process; incidents and adverse events; and HEPMA aspirations (pre) changed to HEPMA implementation (post). Interview topic guide confirmation was achieved by credibility review by research team members and by use of a pilot interview.

All interviews were completed by the principal investigator, an experienced UK hospital pharmacist who had undertaken qualitative interview training. The interviews occurred at a location and time convenient to the interviewee and all were conducted in a private office. The interviews were completed February to August 2013 (pre) and April to June 2015 (post). A deliberate gap of 6 months after implementation completion was left to allow change process conclusion so that the effect of the implementation was captured [26]. The principal investigator used a mixture of key questions and associated probing to ensure all relevant topics were covered, whilst permitting flexibility of discussion. The interview format allowed the interviewee to provide their personal opinion of the prescribing and discharge communication process [27]. Interview duration ranged from 14 to 42 min (pre) and 10 to 45 min (post). Interviews were audio recorded with interviewee consent and transcribed verbatim by the principal investigator using a denaturalised style, in which the interview content is recorded but not the manner of the vocalisation [28]. Transcribed data verification was achieved by other researchers reviewing a random 20% sample of transcripts against recordings.

### Data analysis

The principal investigator completed analysis process: interview transcription, interview familiarisation by rereading transcripts, data coding, framework development, framework application, data charting to framework and data interpretation. Independent coding was completed by the other researchers. The principal investigator entered all transcribed information into NVivo 10© software [29]. The use of a theoretical framework to aid data analysis is recommended because it provides helpful organisation of complex assessments [30, 31]. Initially, the framework approach was used [31]. Thereafter, TDF was used to aid analysis of results for behavioural aspects of the prescribing processes. Table 1 is adapted from Cane et al. [23] and provides a list of the domains and associated constructs.

**Table 1:** Theoretical domains framework adapted from [23]

Domain	Domain definition	Example constructs
Knowledge	An awareness of the existence of something	Procedural Knowledge Knowledge of task environment
Skills	An ability or proficiency adapted through practice	Competence Practice
Social/professional role and identity	A coherent set of behaviours and displayed personal qualities of an individual in a social or work setting	Professional role Professional confidence
Beliefs about capabilities	Acceptance of the truth, reliability or validity about an ability, talent or facility, that a person can put to constructive use	Self-confidence Perceived competence
Optimism	The confidence that things will happen for the best or that desired goals will be obtained	Optimism Unrealistic optimism
Beliefs about consequences	Acceptance of the truth, reliability or validity about outcomes of a behavior in a given circumstance	Outcome expectancies Consequences
Reinforcement	Increasing the probability of a response by arranging a dependent relationship or contingency between the response and the given contingency	Rewards Punishments
Intentions	A conscious decision to perform a behaviour or a resolve to act in a certain way	Stability of intentions Stages of change model
Goals	Mental representation of outcomes or end states that an individual wants to achieve	Target setting Implementation intention
Memory, attention and decision processes	The ability to retain information, focus selectively on aspects of the environment and choose between two or more alternatives	Decision making Cognitive overload/tiredness
Environmental context and resources	Any circumstances of a person’s situation or environment that discourages or encourages the development of skills and abilities, independence, social competence, and adaptive behaviour	Resources Critical incidents
Social influences	Those interpersonal processes that cause individuals to change their thoughts, feelings or behaviours	Social pressure Group conformity
Emotion	A complex reaction pattern, involving experiential behavioural, and physiological elements, by which the individual attempts to deal with a personally significant event or circumstances	Anxiety Stress
Behavioural regulation	Anything aimed at managing or changing objectively observed or measured actions	Self-monitoring Action planning

## Results

### Interviewed staff

Demographic information is provided in Table 2. Nineteen individuals were interviewed pre and post implementation, with data saturation achieved in both phases. Due to staff changes only 10 of the 19 who participated in the pre-implementation interview were available for the post-implementation interview. A further nine individuals were therefore recruited for the post-implementation interviews. None of the interviewees had routine use of HEMPA prior to the study. All post-implementation interviewees were familiar and used HEPMA regularly. The interview phase was completed when total population data saturation was achieved, which accounts for the difference in numbers interviewed amongst the professional groups.

**Table 2:** Interviewee demographics

Pre-implementation				Post-implementation		
Profession	Gender	Years	Experience	Profession	Gender	Years
ANP1	F	15–16	Yes	ANP5	F	23
ANP2	F	27	Yes	ANP6	F	15
ANP3	F	13	Yes	ANP7	F	6
ANP4	F	15	Yes	C7	M	2
C1	M	11	Yes	C8	M	2.5
C2	M	9	Yes	C9	M	12
C3	M	15	No	C10	M	17
C4	F	5	Yes	C11	F	7
C5	M	5.5	No	C12	M	10
C6	M	8	Yes	JD4	F	< 1 year
JD1	F	< 1 year	Yes	JD5	F	< 1 year
JD2	F	< 1 year	Yes	JD6	M	< 1 year
JD3	F	< 1 year	Yes	JD7	F	< 1 year
PH1	M	2	Yes	PH7	M	4.5
PH2	M	7	Yes	PH8	F	6.5
PH3	F	13	No	PH9	F	10
PH4	F	5	Yes	PH10	F	6
PH5	F	4	Yes	PH11	M	8
PH6	F	26	Yes	PH12	F	12

*ANP* advanced nurse practitioner, *C* consultant doctor, *JD* junior doctor, *PH* pharmacist

### General staff experience

Interviewees described traditional prescribing system experiences and discussed associated difficulties. The main themes were inpatient chart and immediate discharge letter (IDL) design and discharge communication process, especially time delays between the IDL and the final typed discharge letter. One particular inpatient issue was ascertaining whether a medicine had been administered, which impacted discharge continuation decision.

“it’s not clear what (medicine) has and hasn’t been given.” [PH4]

IDL structure was discussed and insufficient space and lack of specific sections were highlighted,

“There isn’t anywhere to record the patients’ drug allergy status.” [ANP1]

The existing discharge process was described as leading to significant delays,

“so 3 to 4 month delay in getting them (final typed letter) done,” [C1]

In contrast, post-implementation, mainly positive experiences were articulated,

“I think it is really good and I do think it improves like prescribing and administration of drugs for the patients.” [PH10]

Viewing the inpatient chart was described as improved especially the ability to easily read the prescribed medicines,

“So first of all it’s amazing compared to paper prescription charts because it’s legible.” [C8]

IDL improvement was remarked upon by most interviewees,

“It’s just the quality of the letters that are coming out now, is far better than what we had before with the handwritten prescriptions particularly the clinical information, much more detailed and will be much better for the GP.” [C7]

### Future aspirations with HEPMA

Pre-implementation interviews also explored staff expectations of HEPMA. The majority of comments were positive. Improved safety was the most frequent comment, with aspirations for discharge process system improvement.

“I think it (HEPMA) will make us safer and it will improve communication between primary and secondary care.” [C4]

Concern was expressed about correct system use,

“You probably have to be quite careful if you were starting someone on something that it (HEPMA) could come up with a whole range of different doses for somebody…you might want to be careful to pick the right dose…so many options you accidentally click the wrong one.” [JD1]

### Staff behavioural determinants

**Fig. 1:**
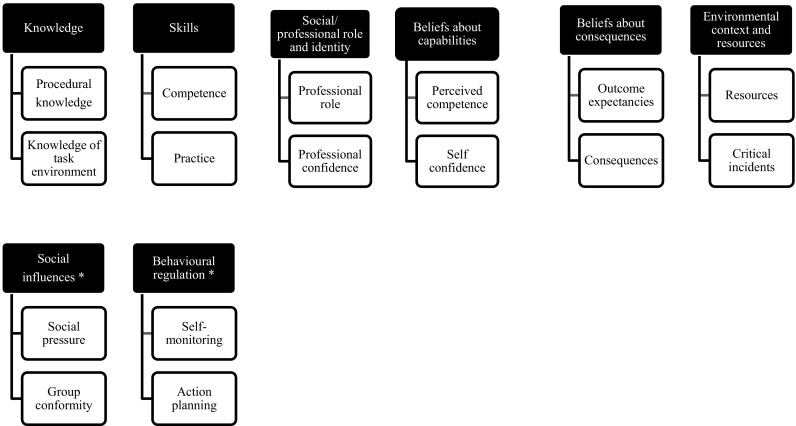
TDF Domains and associated constructs mapped to interview finding. *Domains only applicable post-implementation

Six of the 14 TDF domains emerged pre-implementation; a further two post-implementation. The relevant domains and associated constructs are depicted in Fig. 1.

### Theoretical domains

#### Knowledge

All interviewees described knowing traditional documentation and processes,

“Ok,well the positive side is familiarity…so people understand…how the kardex (inpatient prescription chart) works…“[C1]

A lack of adherence to national guidelines was reported [9],

“Our current drug charts do not easily lend themselves to meeting SIGN requirements for discharge letters…” [C2]

Post-implementation, all interviewees described HEPMA knowledge and processes for inpatient and discharge prescribing. The creation and information on the IDL was described,

“it gives the option…to write exactly what’s happened throughout the patient journey in hospital…medications that have been stopped, again it gives you the allergy status… if GPs should continue it or not, so again it’s very clear.” [PH11]

#### Skills

Interviewees discussed traditional system skills, with legibility a specific concern for inpatient and discharge documentation,

“Quite often it (prescription) is illegible.” [C6]

Post-implementation, junior doctors, pharmacists and ANPs mostly claimed to be skilful system users. A pharmacist described sophisticated system use,

“I feel I can use it quite well,…I know how to like modify things, and can suspend things and resume them…I am probably better at using HEPMA than the doctors are…..” [PH8]

Consultant doctors described varying abilities,

“Yeah, I mean it is quite easy…so when you type the name it gives you the doses for the administration so it’s quite straight forward” [C10]

Whilst another stated he didn’t use the system for prescribing at all,

“My skills are probably limited because I don’t do it.” [C9]

#### Social/professional role and identity

The newer prescribing professions (nurses and pharmacists) focused on professional aspects of prescribing.

“If I’m asked to prescribe something I’ve never prescribed before I won’t do it unless I go and look up the BNF…” [ANP4]

HEPMA implementation was reported to have impacted professional roles to a varying degree as articulated,

“I think probably I’m writing much more on the discharge letters than maybe I would have done previously, maybe prescribing a bit more than previously. I don’t know if that’s the system or just the confidence…I think it has had a positive impact on the pharmacy profession” [PH12]

“I think I spend less time on formal discharge summaries I think that it allows us as a team to get much better information into the GP earlier…” [C7]

#### Beliefs about capabilities

Anxiety when prescribing using the traditional system was reported,

“As a prescriber sometimes I don’t feel very secure, prescriptions may be altered after you have completed them and you don’t know by whom, as they don’t annotate the changes.” [ANP 4]

An increase in prescribing confidence post HEPMA use was described,

“Probably I think my confidence has improved to prescribing and I think that is because I know there is a bit of a safety back up with it” [ANP5]

The exception was consultant medical staff who tended to have more limited use and therefore described themselves as being less competent,

“My skills are in the early stages I would say, as I rely very much on the junior staff.” [C12]

#### Beliefs about consequences

Patient safety concerns and issues with inadequate discharge information provision were discussed,

“There are deep concerns about the safety around about using the paper kardex (inpatient prescription chart), legibility, frequency, recording of administrations, start and finish times and reasons for drug…does lead to medication errors across the boundary into primary care and it also leads to readmissions.” [C1]

Almost all interviewed staff reported receiving GP queries about handwritten IDLs’ information content. The majority of queries related to missing or inaccurate information,

“Always just about please tell me why they are no longer on x,y,z,….Am I meant to be continuing this- it is just lack of clarity on the immediate discharge letter.” [C4]

Post-implementation beliefs about consequences produced the greatest number of comments and therefore divided into sub-themes.

#### Patient safety

Patient safety improvement was articulated by interviewees from all professions, exemplified by,

“I think it’s definitely made a huge difference, a huge improvement in patient safety.” [PH12]

#### IDL quality

IDL quality improvements were frequently cited as a consequence of HEPMA implementation,

“the quality of the discharge prescription has improved because the doctors now use it as a letter to the GP… GPs are getting a lot more information. It’s much easier for the doctors to put in all the medicines that the patient came in on so they are more complete now” [PH10]

#### First and final communication

A change to making the IDL the first and final discharge was described as a consequence of HEPMA,

“the move to having the IDL as the principal discharge document, whereas I felt before that it was the final discharge summary that contained most of the important information…” [C7]

#### HEPMA engagement

An apparent failure by certain consultant doctors to engage with HEPMA was described and this behavior influenced the junior doctor’s perceived pressure when prescribing medicines,

“Well some consultants don’t even use it all…they don’t like it…it leaves a lot of responsibility for the junior members of staff to sort out the medications and it is reliant on just verbal communication from senior doctors telling them to adjust things” [JD4]

#### GP queries

The impact on GP queries was variably described; either having no impact or causing a decrease in calls,

“I’ve had probably one or two queries in the entire time it’s been up…We used to have frequently so maybe two or three phone calls per week from GPs about things.” [ANP5]

#### HEPMA new error types

New error types were suggested to have occurred due to HEPMA implementation as described,

“The drop down boxes it’s very easy for them to pick the first one that comes up when they choose a drug and they don’t actually scroll down to find the correct form for the drug…so it’s a different type of error” [PH9]

#### Environmental context and resources

Pre-implementation interviewees experienced constraints due to existing documentation design and required processes leading to GP information delay. Pressure to complete discharge documentation quickly to hasten discharge which may lead to prescribing errors was described,

“It’s often filled out by a passing doctor trying to facilitate a discharge in a pressurised system.” [C1]

Incident report completion relating to prescribing documentation was only reported by pharmacists, for example,

“…when the wrong patient label was put on a discharge prescription…and it came to that it was actually the patient in the next bed.” [PH1]

Post-implementation, the design and layout of HEPMA inpatient and discharge sections was viewed favourably,

“The layout is very good and I like the box at the bottom of the discharge where it gives you the discontinued drugs and why they have been discontinued” [PH9]

None of the interviewees had completed an incident report regarding HEPMA since implementation. Consensus was that incidents and adverse events were reduced,

“I would guess and I can’t back it up with any figures that it actually has improved the number of incidents and adverse events” [C12]

Social influences and behavioural regulation domains were only applicable to the post-implementation interviews.

#### Social influences

Social pressures to change working practices were described although group conformity was not achieved as described by this doctor,

“Yeah I know other consultants are less comfortable with it, but having used it before…it took me a week or two and then I was back up to speed with it.” [C7]

#### Behavioural regulation

The potential for prescribing errors to occur was raised by several interviewees and actions to avert these were described,

“I think as with any kind of prescribing…you’ve got to get into your own system of checking things and if I prescribe I go back and double check it straight after and yeah I do find the occasional mistake when I’ve put in the wrong strength or put in the wrong frequency but I’ll go and change that right there and then” [PH9]

Table 3 provides a summary of pre and post-implementation findings.

**Table 3:** Summary of findings

Framework		Summary of findings pre-implementation	Summary of findings post-implementation
		Design of inpatient chart, insufficient space on IDL and delays with discharge communication process HEPMA anticipated to improve safety	Improved clarity on inpatient chart and improved quality of IDLs
*TDF Domain*	*TDF Construct*	*Summary of findings pre*-*implementation*	*Summary of findings post*- *implementation*
Knowledge	Procedural knowledge, knowledge of task environment	Staff knew what to do and familiarity described as important, limitations of documentation and processes described	Staff provided detailed descriptions of HEPMA processes and tasks
Skills	Competence, practice	Staff mainly felt competent and ease of access cited as a positive factor, although illegibility described as problematic	ANPs, junior doctors and pharmacists rated themselves as skilful HEPMA users; consultant doctors had varying skill levels
Social/professional role and identity	Professional role, professional confidence	Non-medical prescribers described professional aspect of prescribing	Positive impact on professional role, an increase in confidence described by ANPs and pharmacists
Beliefs about capabilities	Perceived competence, self confidence	Anxiety described due to existing documentation and processes	ANPs, junior doctors and pharmacists all perceived competent; variability with consultant doctors
Beliefs about consequences	Outcome expectancies, consequences	Patient safety a major concern with prescribing errors reported by numerous interviewees, queries from GPs regarding missing or incomplete information frequently related to medicines were reported	Improvement in patient safety, quality of IDL and number of first and final discharge letters, lack of engagement by some consultant doctors and introduction of new error types
Environmental context and resource	Resources, critical incidents	Constraints due to documentation design and time pressures were described, incident reports only completed by pharmacist professional group	Improved design for inpatient and discharge sections, no documentation of a formal incident about HEPMA
Social influences	Social pressure, group conformity	Not applicable	Variability evident amongst practitioners
Behavioural regulation	Self-monitoring, action planning	Not applicable	Process for self-checking developed by some staff

## Discussion

The key findings of this study are reported improvements to staff general experience with prescribing and discharge communication systems and processes post-implementation. The desired outcome of improved safety and enhanced communication between secondary and primary care was described by participants post-implementation. Application of TDF identified behavioural change concepts associated with complex system implementation. The beliefs about consequences domain post-implementation produced the most comments. There were positive descriptions of improved patient safety, IDL quality, enhanced information communication, with GP query reduction in relation to discharge letter content. Negative aspects noted were perceived variability in senior doctor engagement with the electronic system and the creation of a new prescribing error type as a direct consequence of the system. However, behavioural self-regulation was described by some participants to avert prescribing error occurrence. Finally, social pressure was a determinant which contributed to altered working practices for successful system adoption. The findings contribute original knowledge about the perceived benefits and limitations as described by the various staff groups as well as providing insight into behaviour changes adopted by the various professional groups.

Framework analysis was used to identify initial themes then TDF used to analyse behavioural changes. The study findings highlight the complexity of prescribing medicines and communication of discharge information using a HEPMA system from the users’ perspective. Patient safety improvements were claimed to have occurred because of complete prescription legibility, medicine administration accurate documentation, and decision support information availability. This is consistent with previous published literature where electronic discharge letters provide full legibility [11]. IDL quality improvements were frequently quoted with increased clinical and medication information documentation including medicine change information which is consistent with publications demonstrating electronic systems increased dataset compliance [11, 14]. Interviewees reported either unchanged or markedly reduced GP phone calls regarding IDL content which is in keeping with increased quality of information provision and a previous study which demonstrated information enhancement with electronic letters [13]. There were no incident reports completed by post-implementation interviewees and the general consensus was that incidents and adverse events were reduced. A new error type was described, although with no reports of actual patient harm, which is consistent with a previous study that indicated electronic system errors were associated with lower patient harm [16].

Adoption of TDF permitted behaviour change analysis amongst the various professional groups as a consequence of HEPMA implementation. Although some different individuals were interviewed before and after implementation there was consistency in findings irrespective of previous interview. The use of TDF highlighted differences in professional group interplay and this study provides knowledge about behavioural alteration amongst these groups. Consultant medical staff behaviour was reported as the most varied of the studied professional groups; with some consultants refusing to engage with the electronic system, whilst others described sophisticated system use. The implementation of an electronic system may have highlighted an existing disparity in hospital prescribing. Previous research indicated hospital consultants were only responsible for 3.4% of inpatient prescribing activity with several possible causes postulated including availability and culture [32]. The majority of staff deemed themselves as skilful system users. An increase in prescribing confidence with HEPMA was articulated especially by ANPs and pharmacists. Interviewees described adoption of behaviours to ensure GPs received good quality information in the IDLs and resultant process development adopted to achieve this. The associated changes in working systems were instigated as a direct consequence of HEPMA implementation with some consultant teams moving to first and final discharge letters with descriptions of modified processes to achieve this outcome which enables compliance with SIGN 128 vision of changing from IDL to core discharge document [9].

The study strengths include the originality of the work which fills identified literature gaps in relation to hospital staff perspectives before and after electronic prescribing system implementation. The use of TDF theory which enabled a rigorous approach for data analysis about the behavioural aspects of staff involved in the prescribing and discharge communication process. Trustworthiness was achieved by appropriate study design, achieving data saturation and rigorous approach to data analysis to ensure accurate representation of participants’ opinions. Study weaknesses include the variety of experience amongst the different professional groups which may have impacted their responses relating to discharge communication processes. Furthermore, co-existing changes may have occurred during the 20 month time gap between pre and post assessment. Any serious issues raised by staff during the interviews were referred to the appropriate manager by the principal investigator as they involved staff wellbeing and/or patient safety concerns. The study findings are potentially transferable to similar UK NHS organisations and also to other countries with similar healthcare systems. The transferability may be limited dependant on the implemented HEPMA system functionality.

Future process change for discharge information communication should concentrate on the application of a consistent approach amongst the various clinical teams in the production of discharge letters.

## Conclusion

The study findings indicate patient safety and discharge information communication improvement was achieved by HEPMA implementation. Staff clearly articulated complete prescription and IDL legibility, inclusion of more detailed IDL information and enhanced secondary care to primary care information communication. TDF use enabled behaviour change analysis as a consequence of HEPMA implementation for example adoption of behaviours by staff to ensure general practitioners receive good quality discharge information.
